# Exploring spatiotemporal information in a Cherenkov and scintillation photon counting BGO TOF-PET semi-monolithic detector concept

**DOI:** 10.1088/1361-6560/ae2db7

**Published:** 2026-01-08

**Authors:** Seungeun Lee, Ryan Heller, Woon-Seng Choong, Joshua W Cates

**Affiliations:** Applied Nuclear Physics Program, Nuclear Science Division, Lawrence Berkeley National Laboratory, Berkeley, CA, United States of America

**Keywords:** photon counting detector, BGO TOF-PET, Cherenkov detection, analog SiPM

## Abstract

*Objective*. Cherenkov signatures from a bismuth germanate (BGO) crystal open the possibility of establishing BGO as a promising material for time-of-flight positron emission tomography (TOF-PET) detectors, particularly if the first Cherenkov photons can be uniquely timestamped. To maximize the utility of Cherenkov signatures, we employed an optical photon counting detector concept based on a thick, semi-monolithic BGO crystal coupled to a silicon photomultiplier (SiPM) array that provides digital photon timestamps from each SiPM channel. We characterized a prototype detector to demonstrate this concept and explored the use of rich spatiotemporal information of photon transport kinetics. *Approach*. The detector was built using a 42.68 × 2 × 20 mm^3^ BGO crystal and a 16 × 1 array of 2 × 2 mm^3^ SiPMs with a 2.68 mm pitch. A 16-channel low-noise high-frequency signal processing chain with fast comparators generated digital photon signals, which were recorded using waveform digitizers. Three-dimensional (3D) position calibration and first photon delay distribution (FPDD) construction provided the basis for data-driven methods to improve time resolution and estimate the probability of Cherenkov detection for each event. *Main results*. With a sufficient number of SiPM channels and 1.8 ns signal shaping, approximately 77% of events were uniquely timestamped with the first photon. FPDD clearly captured photon arrival properties, parameterized with the Cherenkov and scintillation contributions. A coincidence time resolution with a reference detector of 172 ps full width at half maximum was achieved by FPDD-based correction of 3D position dependence. Parameters investigated for Cherenkov detection probability estimation showed consistent correlation with time resolution. *Significance*. The results demonstrated the feasibility of a photon counting BGO detector for TOF-PET with both promising timing and positioning performance. The abundance of photon information provides a strong basis for further performance gains through data-driven Cherenkov identification and advanced event-by-event corrections.

## 1. Introduction

Bismuth germanate (Bi_4_Ge_3_O_12_; BGO) has reemerged as a promising crystal material for time-of-flight positron emission tomography (TOF-PET). Although BGO was widely used in early non-TOF-PET scanners, it was eventually replaced by faster and brighter cerium-doped lutetium oxyorthosilicate (LSO:Ce) due to its long scintillation decay time (*τ*_d_ ∼ 300 ns) and relatively low light yield (∼8000 photons MeV^−1^), which are undesirable characteristics for precision timing and, consequently, do not enable TOF-PET with BGO using traditional PET instrumentation. However, key studies have highlighted the potential of exploiting prompt Cherenkov emission to enable TOF-PET with BGO (Kwon *et al* 2016, Brunner and Schaart 2017). In addition to its slow scintillation photons, BGO produces approximately 17 prompt Cherenkov photons per photoelectric absorption of a 511 keV annihilation photon, and yields them with high detection probability, owing to its high optical transparency in the ultraviolet region (Roncali *et al* 2019, Gundacker *et al* 2020). As interest in this area has grown, other developments have enabled the practical use of Cherenkov photons in BGO: the development of low noise, high-frequency electronics (Cates *et al* 2018, Cates and Levin 2019, Gundacker *et al* 2020, Krake *et al* 2022) and advances in silicon photomultipliers (SiPMs), particularly in intrinsic single photon time resolution (SPTR) and photodetection efficiency (PDE) (Gundacker *et al* 2023, Merzi *et al* 2023, Lee *et al* 2025). Despite its earlier limitations, the combination of advantages that BGO can offer, such as relatively low material cost, high stopping power and photofraction, no intrinsic radioactivity, and optical transparency to a moderate yield of prompt optical photons, has spurred interest in what may be possible with further advances in detection technologies and techniques.

**Figure 1.**
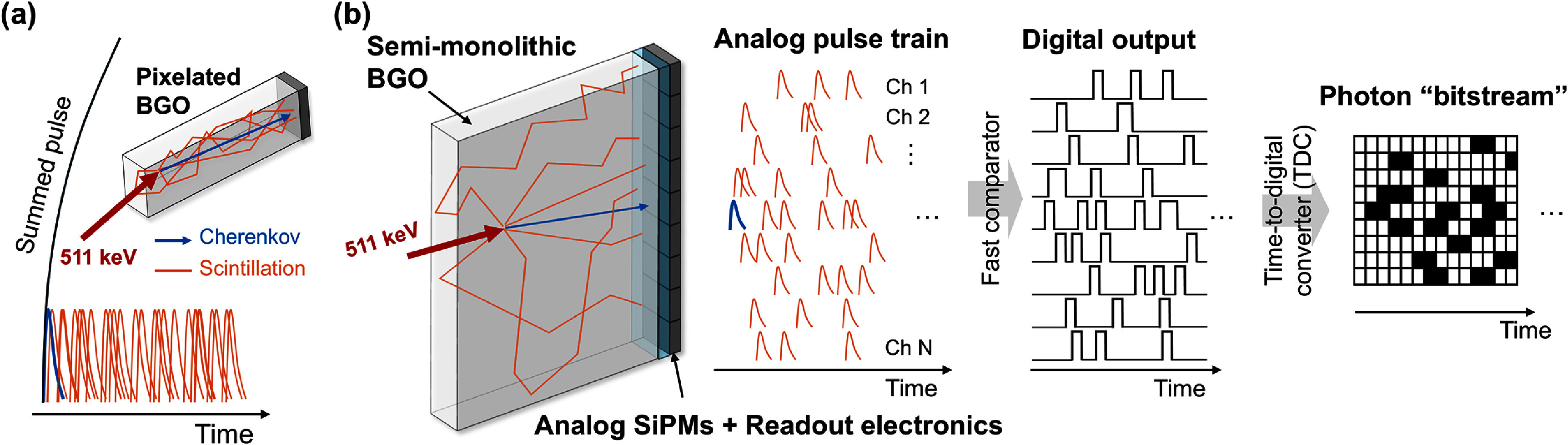
Conceptual illustration of the proposed semi-monolithic BGO Cherenkov and scintillation photon counting detector. Individual optical photons induced by an annihilation photon interaction are read out as a data architecture of photon bitstream by passing through low-noise, high-frequency electronics, comparators, and TDC in order.

In optimizing a 511 keV photon time-of-interaction estimator in detectors with limited Cherenkov photon yield, it is essential to purely extract information from the prompt Cherenkov and early-arriving scintillation photons (Gundacker *et al* 2020). For such detectors, obtaining a ‘digital’ timestamp of the first photon is highly beneficial for enhancing the time resolution, compared to ‘analog’ timestamping the full signal. This benefit is further pronounced for current SiPM technologies with SPTRs of a few tens of picoseconds. (Gundacker *et al* 2023). If the optical photons can also be labeled as ‘Cherenkov’ or ‘scintillation’, then that additionally benefits the achievable timing performance (Loignon-Houle *et al* 2023). However, in standard detector configurations consisting of a high-aspect-ratio crystal pixel coupled to a SiPM pixel, timestamps obtained using a simple leading-edge discriminator are influenced by the detection of following scintillation photons, leading to inclusion of their information in time pickoff from the summed pulse (figure 1(a)). Some approaches that extract Cherenkov information from the sampled waveforms include deconvolving the single photon response to recover digital photon timestamps (Ota and Ote 2024) and implementing neural network on the rising slope (Loignon-Houle *et al* 2025). Another approach estimates the population of Cherenkov by leveraging its correlation with the photon arrival density at the rising edge. Here, events are categorized by the signal rise time to perform time skew correction for each category, thereby improving time resolution (Kratochwil *et al* 2020, 2021a) and enabling multi-kernel TOF techniques to enhance reconstructed image quality with BGO (Efthimiou *et al* 2021, Nuyts *et al* 2023). This event categorization can also be performed by measuring early timestamps using single-ended (Yi *et al* 2025) or dual-ended readout (Kwon *et al* 2019, Kratochwil *et al* 2025). It is also important to identify the earliest photons with the shortest and least complex travel paths, thus carrying the most precise timing information. Several studies on surface treatments demonstrated that signals without the contribution of photon reflection to the transit time dispersion exhibited better timing precision of the fast photons, both in pure Cherenkov emitters and BGO crystals (Dolenec *et al* 2016, Kratochwil *et al* 2021b, Lee *et al* 2023).

Our proposed detector concept for extracting the unique first photon timestamps of BGO is to count every optical photon using a monolithic crystal coupled to an array of SiPMs (Cates *et al* 2024). As shown in figure 1(b), the large, continuous volume of the (semi-)monolith allows the dispersion of the optical photons and thereby creates both spatial and temporal sparsity in photon arrivals at the SiPM pixels. This configuration is equivalent to sampling the event with multiple channels, providing spatiotemporal information on individual photons, and increasing the likelihood of uniquely extracting information from early detected photons. Another potential advantage of employing a crystal monolith is the reduced transit time variance for the photons emitted toward the SiPMs, due to a lower likelihood of crystal and reflector surface interactions, which can decrease the randomness of photon trajectories and the loss probability of early-arriving photons. Additionally, a thin optical bandpass filter is placed between the crystal and SiPMs to efficiently suppress the propagation of external optical crosstalk throughout the crystal volume. It selectively transmits scintillation and Cherenkov photons while absorbing crosstalk photons generated during the SiPM avalanche process (Lee *et al* 2024). The crosstalk suppression afforded by this bandpass filter allows SiPM arrays to be operated at high overvoltage and at room temperature. Benefiting from the excellent SPTR of the state-of-the-art SiPMs and a low-noise high-frequency (LNHF) signal processing chain, trains of precisely resolved single photon pulses are produced for each SiPM channel. The analog pulse trains are digitized by a fast low-voltage differential signaling (LVDS) comparator with the threshold at the midpoint of the single photon pulse height. Ideally, a single photon is represented as a binary pulse with a fixed pulse width (a few ns), fixed pulse height, and timestamp at the rising edge. Although some piled-up optical photons may lose their timestamps, the number of pileups can still be derived from the time-over-threshold (TOT) of the photon pulse. An envisioned readout architecture involves feeding the photon ‘bitstreams’ directly to a multi-hit time-to-digital converter (TDC) to count the photons and process their timestamps for timing and positioning estimation. The intrinsic capability of monolithic detectors to provide three-dimensional (3D) positioning of 511 keV events further offers the potential to correct for the transit time variations depending on the interaction position.

In this work, we evaluated a prototype Cherenkov and scintillation photon counting detector configured with a semi-monolithic BGO crystal read out by a 16 × 1 array of SiPM pixels. The semi-monolithic slab design guides the optical photons along one dimension, allowing for a simplified position calibration (Zhang *et al* 2021, Freire *et al* 2022, Kuhl *et al* 2024). The number of channels was chosen based on our previous study, which showed good optical sparsity using a 4 × 4 SiPM array and a monolithic crystal (Cates *et al* 2024). We employed small 2 × 2 mm^2^ SiPMs to fully leverage their excellent SPTR and to achieve a fixed 2 mm pixelation along the crystal-stacking direction. We first characterized the optical photon transport properties by correlating photon arrivals with the 3D position of interaction through 1D position calibration. We then assessed the achievable time resolution and explored the parameters that may correlate with the likelihood of Cherenkov detection in each event.

## 2. Materials and methods

### 2.1. Detector setup

**Figure 2.**
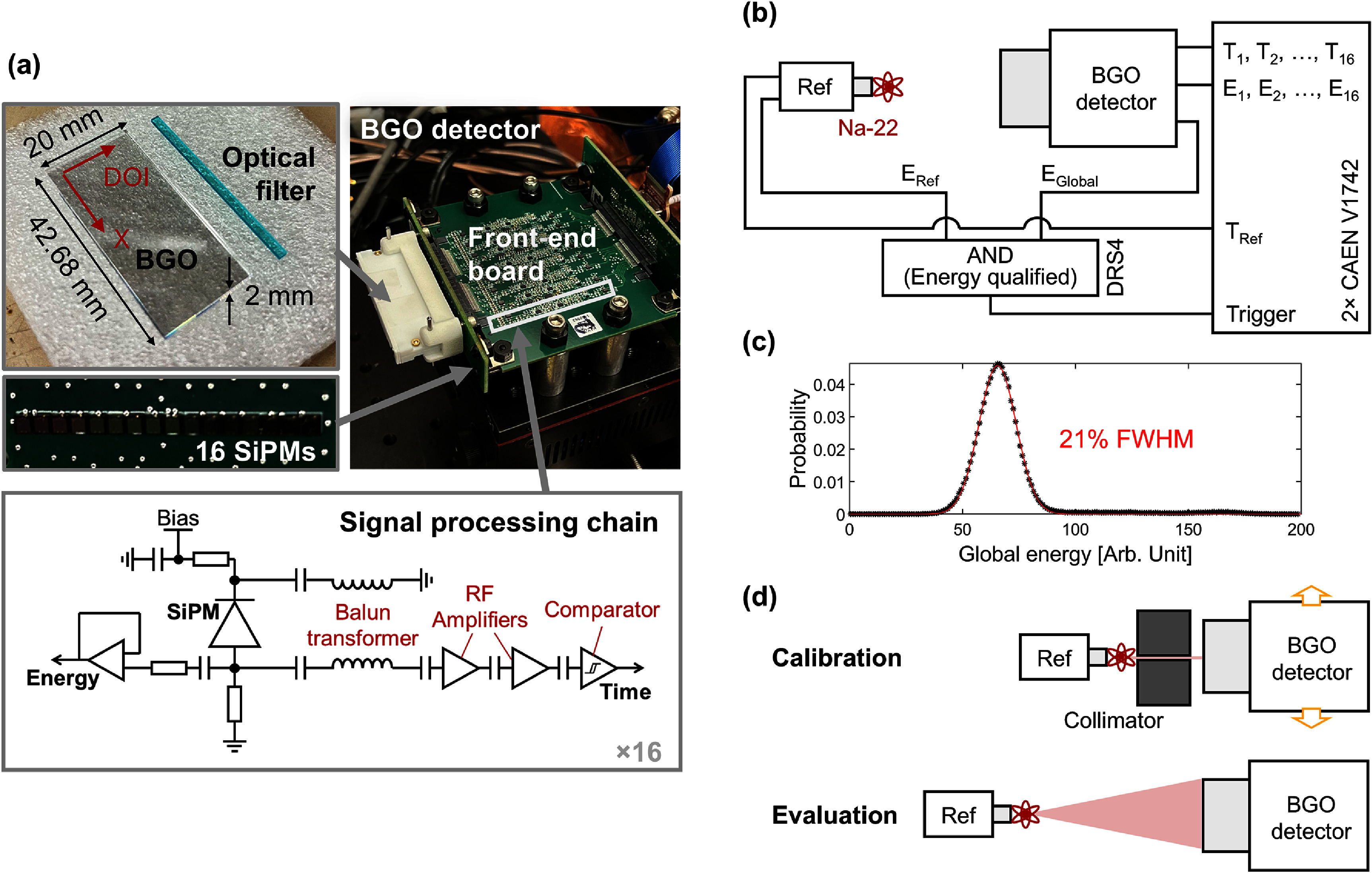
Experimental setup for photon counting detector characterization. (a) Detector components, (b) data acquisition scheme for coincidence measurement, and (c) calibration and evaluation setup.

The prototype detector consisted of a 42.68 × 2 × 20 mm^3^ BGO crystal (Epic Crystals) with all sides mechanically polished, a 42.68 × 2 × 1 mm^3^ optical bandpass filter (BG40; Schott), and a SiPM board with a 16 × 1 array of 2 × 2 mm^2^ SiPMs (AFBR-S4N22P014M; Broadcom) with a 2.68 mm pitch, as shown in figure 2(a). The fill factor of the crystal covered by the SiPM active area was hence 75%. Each interface was optically coupled using Meltmount (*n* = 1.58; Cargille). A custom Teflon detector casing was used as both a reflector and mechanical support for the crystal-SiPM board assembly.

The assembly was mounted on the edge of a front-end board that processes the 16 analog SiPM signals for photon counting. We implemented a LNHF readout scheme that offers an excellent signal-to-noise ratio for single photon detection, using a balun transformer and two-stage radiofrequency (RF) amplifiers in series (Cates and Choong 2022). A fast LVDS comparator (MAX40025; Maxim Integrated) was added at the end of the chain, configured with the threshold at 60 mV, which corresponded to half-height of the analog single photon pulse after shaping and processing. An energy readout chain was also implemented using a low-power (∼3 mW) voltage buffer (OP890; Texas Instruments) to measure the signal amplitude of each channel. The energy signals were split between individual channel energy readout and a global energy channel for triggering and event qualification. Notably, the circuit topology was compact enough to accommodate all 16 channels and their components behind the SiPM array, enabling edge-mounted slab detector stacking in future scale-up implementations (even in an unoptimized layout shown in figure 2(a)). A backplane board was also fabricated to interface the readout channels, SiPM bias voltage, and active component voltages.

Figure 2(b) shows the readout setup for coincidence measurements. A reference detector was assembled using a 2 × 2 × 3 mm^3^ LYSO:Ca,Ce (Taiwan Applied Crystals) coupled to a 4 × 4 mm^2^ SiPM (AFBR-S4N44P014M; Broadcom) and read out with the same timing signal processing chain used for the BGO detector. All SiPMs were biased with an overvoltage of 16 V, which is the lowest voltage that achieves superior timing performance (Lee *et al* 2025). An external DRS4 evaluation board (Paul Scherrer Institute) received the global energy signals of both the BGO and reference detectors and generated coincidence trigger signals by applying AND logic at the 511 keV qualifying lower threshold. The global energy resolution of the BGO detector was measured to be 21% full width at half maximum (FWHM) (figures 2(c)), and a 511 keV event selection window was set to peak ± 2 FWHM. The triggers were fed into two daisy-chained V1742 digitizers (CAEN), one running at a sampling rate of 5 GSa s^−1^ for the fast time signals and the other at 750 MSa s^−1^ for the energy signals, enabling waveform sampling time windows of 204 ns and 1.3 *μ*s, respectively. The time resolution of the single reference detector was quantified as 45 ps in FWHM using this readout scheme. Photon timestamps were picked off by applying a leading-edge discrimination threshold to the sampled fast signal waveforms, which were interpolated using a cubic spline to mitigate the quantization error. The signal amplitude of each SiPM channel was quantified by integrating the energy signal waveforms. The temperature was consistently maintained at 20 °C throughout the measurements using a Peltier cooler (Thorlabs) and temperature-controlled airflow. Note that in this experimental setup, time pickoff occurs on the analog SiPM signal with the comparators on the readout board of the BGO photon counting detector. We digitize the digital signals from the comparators. Thus, we are using the V1742 digitizer as a TDC, not capturing raw timing waveforms from the SiPMs.

**Figure 3.**
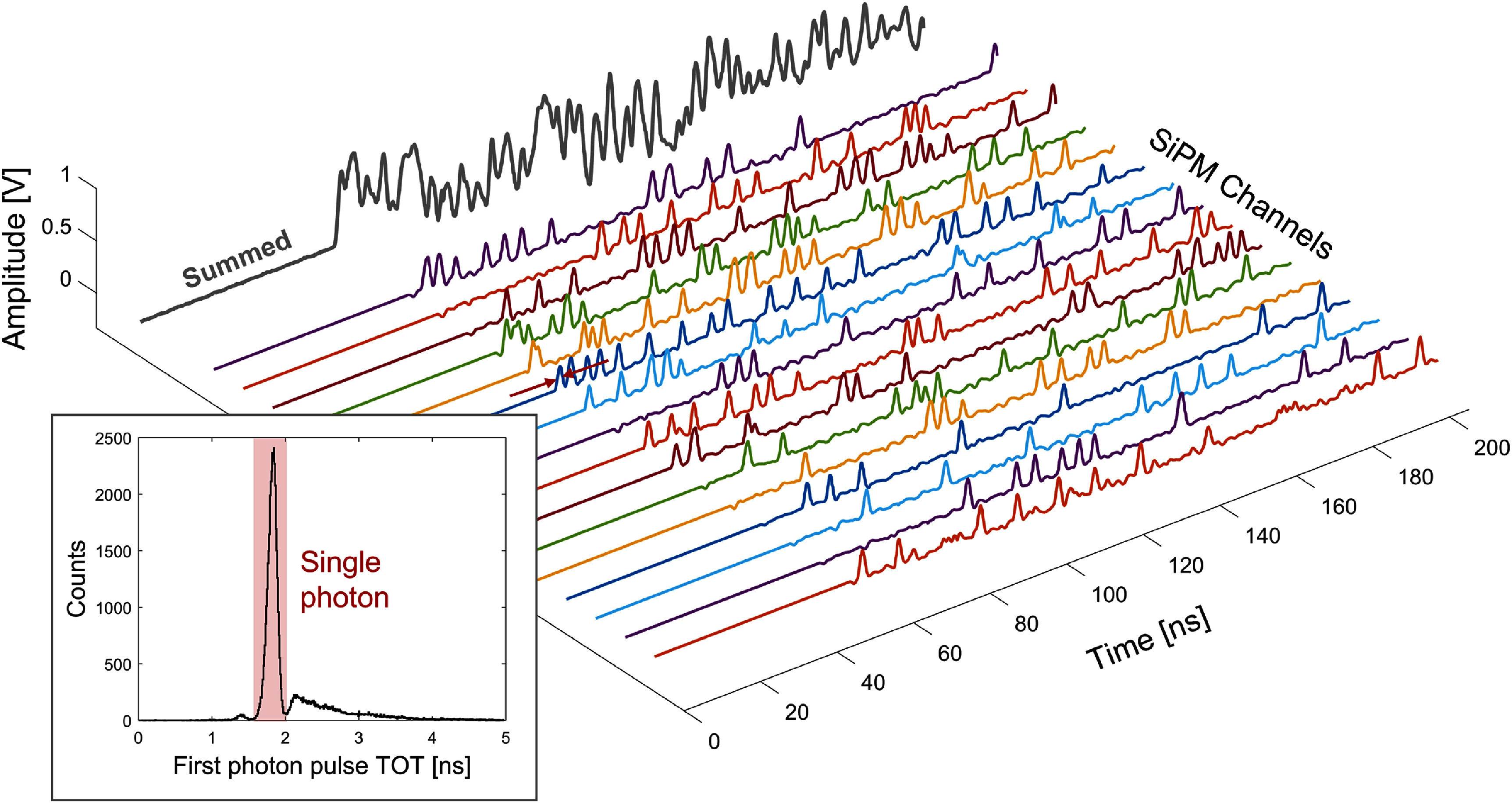
An example 16-channel photon counting output for a 511 keV interaction within the BGO crystal. The probability of the first photon being a single resolved photon was derived from the pulse TOT histogram.

The combination of LNHF electronics and the readout setup produced the photon counting outputs from 16 individual timing channels, as shown in figure 3. Each pulse with a width of 1.8 ns represents a single photon response, while the pileup of photon arrivals and/or optical crosstalk results in wider pulses. The clear dispersion of the majority of photons from the BGO implies good optical sparsity in the detector configuration and hence the potential to resolve most of the photon arrivals. By quantifying the TOT of the first digital photon pulse for each 511 keV interaction, which links the number of photons for the comparator outputs, the probability of the first photon being resolved as a single photon was measured to be 69%. The first digital pulses greater than one photon equivalent width represent pileup due to multiple optical photon arrivals and direct crosstalk.

### 2.2. SPTR measurement

Before coupling the crystal, we measured the SPTR of the 16-channel SiPM readout chain using a picosecond-pulse laser that produces 408 nm photons at a repetition rate of 100 kHz, with a pulse width of 24 ps FWHM. The SiPM board was irradiated with the laser using a neutral density filter and an optical diffuser to generate microcell avalanche signals in the single photon counting regime for each channel. Pileup events (e.g. multiple avalanches or optical crosstalk) were rejected based on the photon pulse TOT distributions. The SPTR was measured as the FWHM of the time delay distribution of the single photon pulse from the laser tag pulse, both sampled by V1742 digitizers. The contribution of the laser signal time jitter (24 ps FWHM) was subtracted in quadrature. A detailed description of the SPTR measurement setup can be found in the literature (Nemallapudi *et al* 2016, Cates *et al* 2024). We also quantified the electronic skew to correct for relative timing offsets introduced by variations in signal path lengths across the readout channels.

### 2.3. Crosstalk probability measurement

The population in the region with TOT greater than a single photon pulse width (TOT histogram in figure 3) consists of pileups due to multiple photons and crosstalk. To estimate the contribution of crosstalk, we measured the crosstalk probability of the fully assembled detector. The SiPM timing signals were randomly captured with a 204 ns time window using a software triggering mode of the digitizers, without any radioactive sources, yielding negligible scintillation photon pileup probability. For each channel, we generated a TOT histogram of captured pulses and measured the portion of the single photon pulse. While the TOT histograms obtained from 511 keV interaction and SPTR measurement include externally induced optical pileup, this configuration ensures the pure effect of optical crosstalk from the dark noise of the SiPMs.

### 2.4. Event positioning

1D position calibration of the BGO detector was performed along the *X* direction using the acquisition setup depicted in figure 2(d). A 511 keV fan-beam was created by placing a 3.7 MBq Na-22 point source and the reference detector behind a 1 mm slit opening of a 10 cm thick collimator, oriented perpendicular to the top surface of the BGO crystal at 5 mm distance. The detector was moved from −20.5 mm to 20.5 mm in 1 mm steps along the *x* axis using an automated platform that controlled both the translational stage and digitizers. Approximately 20 000 511 keV events were collected for each *X* irradiation for 3 h. A convolutional neural network (CNN) was designed as a series of two convolutional blocks, a 50% dropout layer, and two fully connected layers, whereas a convolutional block consisted of a convolution, a batch normalization, a rectified linear unit, and a 2 × 1 pooling layer. The network was trained to estimate the *X* position of interaction using the 16 × 1 energy signal amplitudes as input with a minimal number of layers and neurons. The positioning bias of the CNN was recorded for each *X* position and used to correct for uncollimated events.

Instead of performing a separate calibration along the depth-of-interaction (DOI) direction, we used a max-to-mean (MM) ratio of the 16 energy signal amplitudes to estimate DOI, provided that simple analytic metrics can still achieve adequate accuracy (Gonzalez-Montoro *et al* 2017). A deeper DOI introduces more concentrated reception of the photons in the SiPM channels closer to the interaction point, yielding a higher MM ratio. For each X position dataset, events were sorted by their MM ratio and categorized into four DOI bins with equal counts. The effective centers of the DOI bins were inferred to be 1.29, 4.28, 8.43, and 15.44 mm based on attenuation of the incoming flux governed by the Beer–Lambert law. The MM boundary values were recorded and later used to assign the DOI bins for events collected during the subsequent measurements. Accordingly, the 511 keV interaction positions were categorized into 42 (*X*) × 4 DOI virtual voxels within the crystal volume.

### 2.5. First photon delay distribution (FPDD) assessment

Inspired by Borghi *et al* (2016) and van Dam *et al* (2013), we correlated the first photon arrival time and position with the 511 keV interaction position by constructing the probability density function (PDF) of the first photon timestamp delay relative to the reference detector time signal, referred to as the FPDD. The FPDD PDF, denoted as ${f_{{\text{FPDD}}}}$, over time $t$ given an interaction time $\theta $, was defined for each combination of the crystal voxel and SiPM pixel. Therefore, we obtained 42 (*X*) × 4 (DOI) × 16 (SiPM) FPDDs in total. Each FPDD was approximated as the sum of Cherenkov ${f_{\text{c}}}$ and scintillation ${f_{\text{s}}}$ components, convolved with the Gaussian impulse response of the detection system $g$:

\begin{equation*}{f_{{\text{FPDD}}}}\left( {t|\theta } \right) = \left( {{f_{\text{c}}}\left( {t{\text{|}}\theta } \right) + {f_{\text{s}}}\left( {t{\text{|}}\theta } \right)} \right)*g\left( t \right).\end{equation*}

In detail, based on the prompt nature of Cherenkov and the double exponential behavior of scintillation emissions, the profiles ${f_{\text{c}}}$, ${f_{\text{s}}}$, and $g$ were parameterized using the Dirac delta function $\delta $ and Heaviside step function ${{\Theta }}$ as follows (Gundacker *et al* 2018, Loignon-Houle *et al* 2023):

\begin{equation*}{f_{\text{c}}}\left( {t{\text{|}}\theta } \right) = C \cdot \delta \left( {t - \theta } \right)\end{equation*}

\begin{equation*}{f_{\text{s}}}\left( {t{\text{|}}\theta } \right) = {{\Theta }}\left( {t - \theta } \right) \cdot \rho \cdot \frac{{{{\text{e}}^{ - \left( {t - \theta } \right)/{\tau _d}}} - {{\text{e}}^{ - \left( {t - \theta } \right)/{\tau _r}}}}}{{{\tau _d} - {\tau _r}}}\end{equation*}

\begin{equation*}g\left( t \right) = \frac{1}{{\sqrt {2\pi } \sigma }}{{\text{e}}^{\left( { - {t^2}/2{\sigma ^2}} \right)}}.\end{equation*}

The interaction time $\theta $ encompasses 3D position information of the 511 keV interaction, including the flight time from the crystal surface entrance to the interaction point and the transit time of the emitted prompt photons. It also accounts for the electronic skew, which was measured from the laser setup in section 2.2. The parameter $C$ represents the relative amplitude of the Cherenkov profile, while $\rho $ represents that of scintillation. The decay time ${\tau _{\text{d}}}$ and rise time ${\tau _{\text{r}}}$ incorporate not only the intrinsic emission properties of the scintillator but also the transport dynamics of the scintillation photons, which are position-dependent. The set of ${\tau _{\text{d}}}$ and ${\tau _{\text{r}}}$ can be modeled for multiple scintillation process components and weighted by the respective $\rho $. However, modeling a single scintillation component was sufficient to achieve an acceptable goodness of fit. $\sigma $ denotes the temporal standard deviation of the single photon detection response. While it can be approximated by the SPTR of the readout chain, it also includes contributions from Cherenkov transit jitter in the BGO crystal. All the parameters were estimated as functions of the interaction voxel and the SiPM pixel pair by fitting the corresponding FPDD.

#### 2.5.1. Electronic time skew derived from FPDD

To validate the accuracy of estimating the parameter $\theta $ and further explore the usefulness of FPDD, we derived the electronic time skew of each SiPM channel from the FPDDs, utilizing the detector’s DOI estimation capability, and compared the values with those measured from the laser setup in section 2.2. For SiPM *i*, we selected the crystal voxel *V*_i_ in the top DOI layer, located closest to the center of the channel (e.g. crystal voxel 1 for SiPM channel 1, illustrated in figure 6(a)). This voxel-channel pair is chosen because the top DOI voxels populate a thin layer of the front of the crystal, where the perpendicular transit time variance between the Cherenkov photons initially propagating towards and away from the photosensor is minimized. The top DOI voxels were chosen because the interactions were populated within this thin layer, which is beneficial for precise measurement of the perpendicular travel time toward the SiPMs. Consequently, the time skew for channel *i* was estimated as the parameter $\theta $ of the corresponding (*V_i_, i* ) pair derived from the FPDD.

### 2.6. Time resolution

An uncollimated dataset for time resolution evaluation was acquired by uniformly irradiating the BGO detector using the setup depicted in figure 2(d). A Na-22 point source was mounted on top of the reference detector to accept a wide angular range of paired annihilation photons. The source was positioned 23 cm from the top surface of the BGO detector, aligned along the central axis. A total of 61 000 photopeak events at 511 keV were collected for analysis from a 40 h acquisition.

We measured the time resolution of the BGO detector incorporating the FPDDs to correct for the 3D position dependency. *X* and DOI of each event were positioned using the trained CNN and MM metric, respectively, and the corresponding crystal voxel was assigned to retrieve the $\theta $ parameter from its FPDD. The event timestamp was determined as the earliest among the 16 digital leading edges, in other words, the very first photon arrival (*T*_1_). This approach outperformed other analytical timing estimators, such as averaging the first few photon arrivals or a weighted average, due to the contribution of the prompt Cherenkov signatures to the time precision. The 3D position skew was corrected by simply subtracting the FPDD-derived $\theta $ from the photon timestamp. To reject the false leading photon pulses (e.g. SiPM dark noise) in event timestamping, we applied a noise filtering algorithm inspired by the trigger logic of Philips Digital Photon Counter (Schaart *et al* 2016). The algorithm validated a leading edge as the event timestamp only if other channels registered additional photon pulses within a 5 ns coincidence window. Otherwise, the windowing logic was iteratively applied to subsequent leading edges until validation. The time delay distribution of the BGO detector relative to the reference detector was fitted with a sum of a single Gaussian and an exponentially modified Gaussian to model the arrival profiles of Cherenkov and scintillation, respectively:

\begin{equation*}y = {A_f}{{\text{e}}^{ - {{\left( {x - {\mu _f}} \right)}^2}/\left( {2\sigma _f^2} \right)}} + {A_s}\frac{{{\lambda _s}}}{2}{{\text{e}}^{\frac{{{\lambda _s}}}{2}\left( {2{\mu _s} + {\lambda _s}\sigma _s^2 - 2x} \right)}}\left(1 - {\text{erf}}\left( {\frac{{{\mu _s} + {\lambda _s}\sigma _s^2 - x}}{{\sqrt 2 {\sigma _s}}}} \right)\right){ }{\text{}}.\end{equation*}

The parameters $A$, $\mu $, $\sigma $, and $\lambda $ represent gain, peak position, standard deviation, and tail delay, respectively (Nemallapudi *et al* 2016). The subscripts $f$ and $s$ denote fast and slow components, respectively, and erf corresponds to the error function.

### 2.7. Cherenkov detection probability-related parameters

Leveraging the photon counting detector’s ability to identify multiple photons and localize interaction positions, we explored several parameters potentially correlated with the probability of Cherenkov photon detection in individual events.

1) Events exhibiting a higher temporal density of early photon arrivals are more likely to contain Cherenkov signatures. Among the 16 leading-edge timestamps, the time difference between the first and second photon arrivals was measured and denoted as *T*_2_–*T*_1_. This metric is analogous to the rise time of single-channel BGO signals (Kratochwil *et al* 2020) and to the timestamp difference measured by a dual-channel segmented SiPM (Yi *et al* 2025). In contrast, our configuration provides single-photon-level timestamps across spatially dispersed channels.
2) To incorporate the directional bias of the Cherenkov photon emission from the gamma-ray interaction, we defined a parameter Δ_Ch_ as the channel difference between the SiPM pixel located directly beneath the estimated interaction position and the pixel that detected the first photon. A smaller Δ_Ch_ implies a shorter optical path and fewer reflections, suggesting a higher likelihood that the first photon is Cherenkov.
3) As introduced in section 2.5, the FPDDs were fitted with a composite of fast (Cherenkov) and slow (scintillation) components. Looking up the FPDD of a pair between the event position and the first photon detected channel, the probability of the first photon to be Cherenkov, *p*_Cheren_, was computed from the relative contribution of the Cherenkov component to the total probability at the first photon timestamp ($t = {T_1}$):

\begin{equation*}{p_{{\text{Cheren}}}} = { }{f_{\text{c}}}\left( {{T_1}{\text{|}}{\theta _{{\text{ref}}}}} \right)/\left( {{f_{\text{c}}}\left( {{T_1}{\text{|}}{\theta _{{\text{ref}}}}} \right) + { }{f_{\text{s}}}\left( {{T_1}{\text{|}}{\theta _{{\text{ref}}}}} \right)} \right)\end{equation*}

where ${\theta _{{\text{ref}}}}$ denotes the reference detector signal timestamp.

For each of the three candidate parameters, events were sorted and grouped into 5 bins with an equal number of events. We measured the ratio between FWTM and FWHM of the time delay distribution for each bin as a metric of the distribution shape.

## 3. Results

### 3.1. SPTR measurement

**Figure 4.**
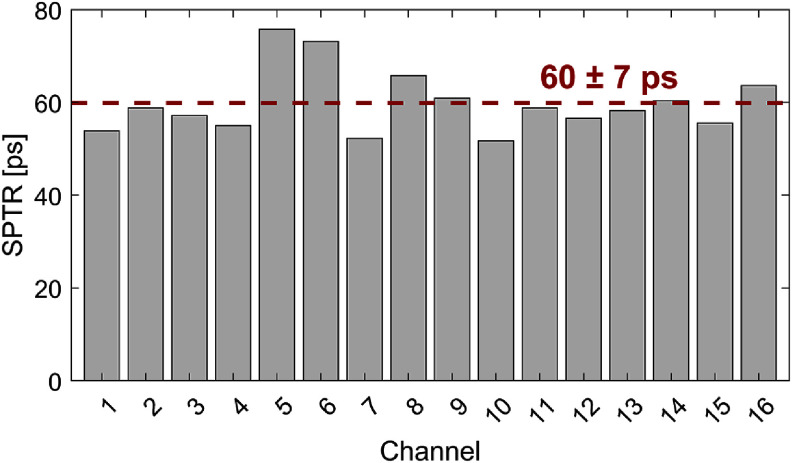
Measured SPTR per channel.

The SPTR of the 16-channel electronics board, combined with the readout setup, is presented in figure 4. The measured SPTR values include effects of the intrinsic time jitter of the 2 mm SiPM device, noise contribution of the electronics chain, and the time pickoff uncertainties associated with digitized comparator signal output. The average SPTR across the 16 channels was 60 ± 7 ps FWHM.

### 3.2. Crosstalk probability

The crosstalk probability across the 16 SiPM channels was quantified to be 7.8% ± 1.6%, representing the probability of a single SiPM trigger being accompanied by direct crosstalk or early external crosstalk. Compared to the crosstalk probability of the SiPM device reported in the datasheet (Broadcom 2023), which is 30% at 16 V overvoltage, the pileup was substantially reduced by the optical bandpass filter, which refracts and absorbs the crosstalk photons (Lee *et al* 2024).

### 3.3. Event positioning

**Figure 5.**
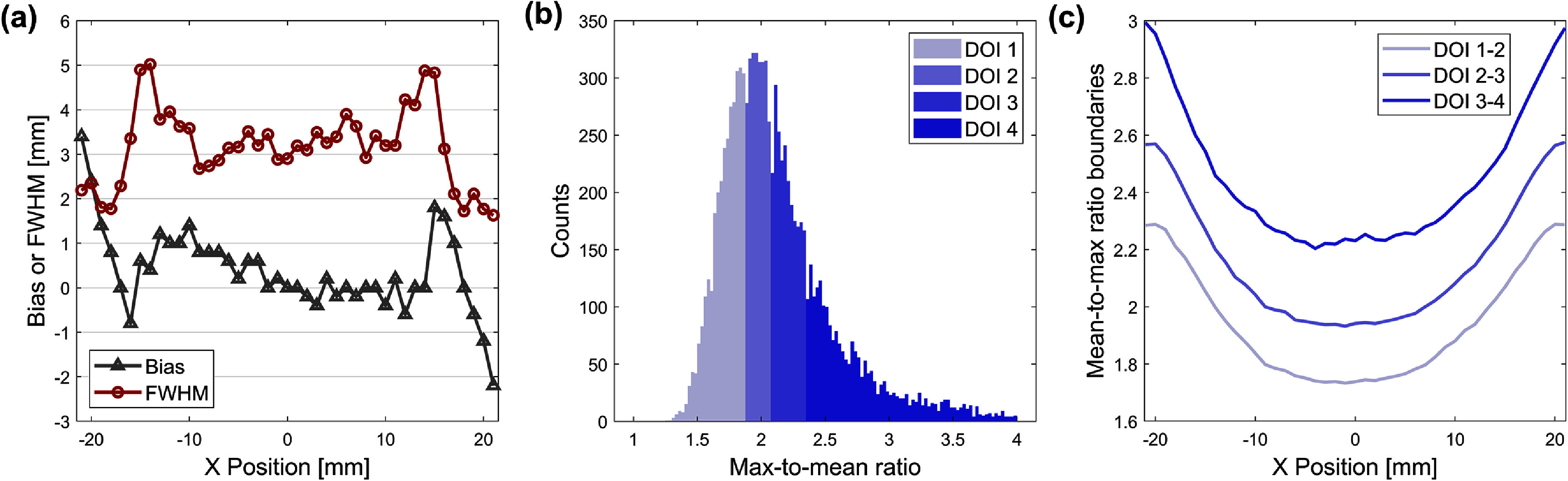
Position calibration results. (a) Bias and resolution of CNN-based X positioning as functions of true X. (b) MM ratio distribution example of X = −10.5 mm dataset with sorting the events populating the counts evenly across four DOI bins. (c) MM ratio boundaries for DOI bin assignment for each X position.

*X* and DOI calibrations of the 511 keV interactions were performed using the fan-beam irradiation dataset. Figure 5(a) shows the *X* positioning performance of the CNN, yielding a global resolution of 3.17 mm FWHM and a global absolute bias of 0.69 mm throughout the crystal volume. The larger bias and worse resolution at the edge regions were attributed to optical photon reflections, which deteriorate the uniqueness of the light pattern. Figure 5(b) shows the distribution of the MM ratio for an example of *X* = 10.5 mm, which was used to define the boundaries for DOI segmentation, plotted in figure 5(c). Higher MM ratios observed at the edge region indicate more localized photon arrivals near the SiPM pixels.

### 3.4. FPDD

**Figure 6.**
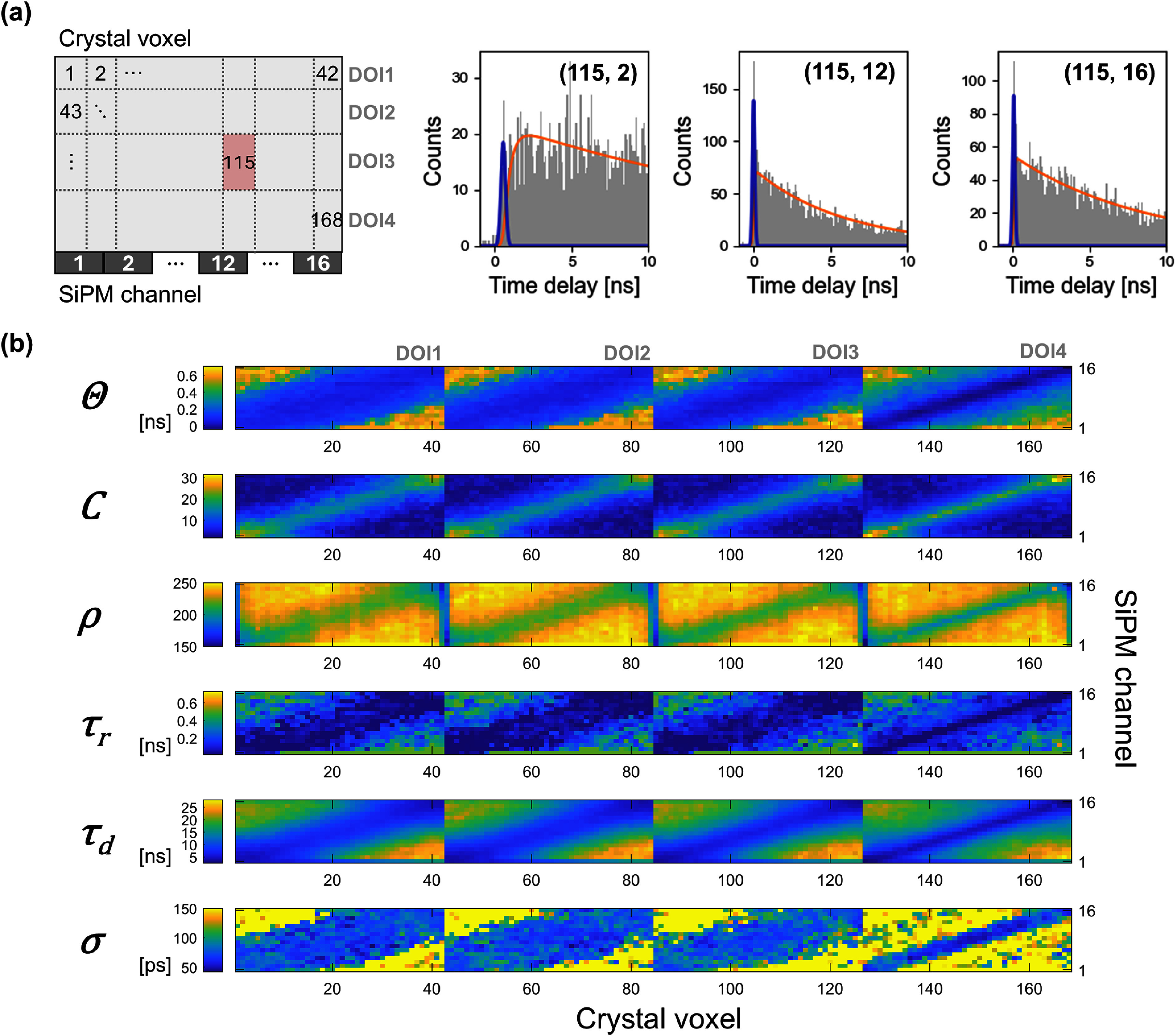
FPDD constructions for crystal voxel and SiPM channel combinations. (a) Example FPDDs and fitted curves for voxel 115 (X = 10.5 mm, DOI bin = 3) combined with SiPM channels 2, 12, and 16. Left figure not drawn to scale. (b) Maps of the fitted parameters with the indexing rule depicted in (a).

FPDD PDFs were constructed for each pair of interaction voxel and SiPM channel using the calibration dataset and were fitted with the model in (1). Example FPDDs for a fixed voxel with three different SiPMs are shown in figure 6(a), where the Cherenkov component is most prominent for the SiPM directly under an interaction voxel and gradually diminishes for more distant SiPMs. The strong dependence of FPDD shape on relative positions between the interaction position and SiPMs is visualized by mapping the fitted parameters (figure 6(b)). The maps are clearly segmented into four along the voxel axis, where each segment corresponds to a DOI bin.

For all parameters, a clear relationship among the SiPM channels and their nearest voxels was observed along the diagonal line. Small $\theta $ values on this line indicate short photon travel paths towards perpendicular SiPMs, while $\theta $ increased along *X* more steeply for edge channels than for those near the center. Across the DOI bins, a segment with a deeper DOI showed this orthogonal pattern with higher contrast and narrower width, reflecting both the reduced Cherenkov transit time to nearby channels and steeper distance gradients across the SiPM pixels. The estimated $\theta $ spanned from 0 to 712 ps, implying the importance of the position-based correction for the time resolution. The parameter $C$, representing the Cherenkov yield, was higher for the SiPMs closer to interaction voxels due to their larger solid angle and shorter photon path length. On the other hand, the scintillation yield $\rho $ showed an opposite trend because it complements $C$ in forming the overall PDF ${f_{{\text{FPDD}}}}$. ${\tau _{\text{r}}}$ and ${\tau _{\text{d}}}$ also varied with distance because they encompass not only the scintillation emission profile but also the travel length that induces variance in detectability. $\sigma $ remained relatively uniform in regions where Cherenkov yields were significant, aligning with SPTR values quantified in section 3.1, which were convolved with the optical transit time profile of each voxel. Estimating $\sigma $ was less reliable in regions with small $C$, where the Cherenkov peak was obscured by the scintillation.

#### 3.4.1. Electronic time skew derived from FPDD

**Figure 7.**
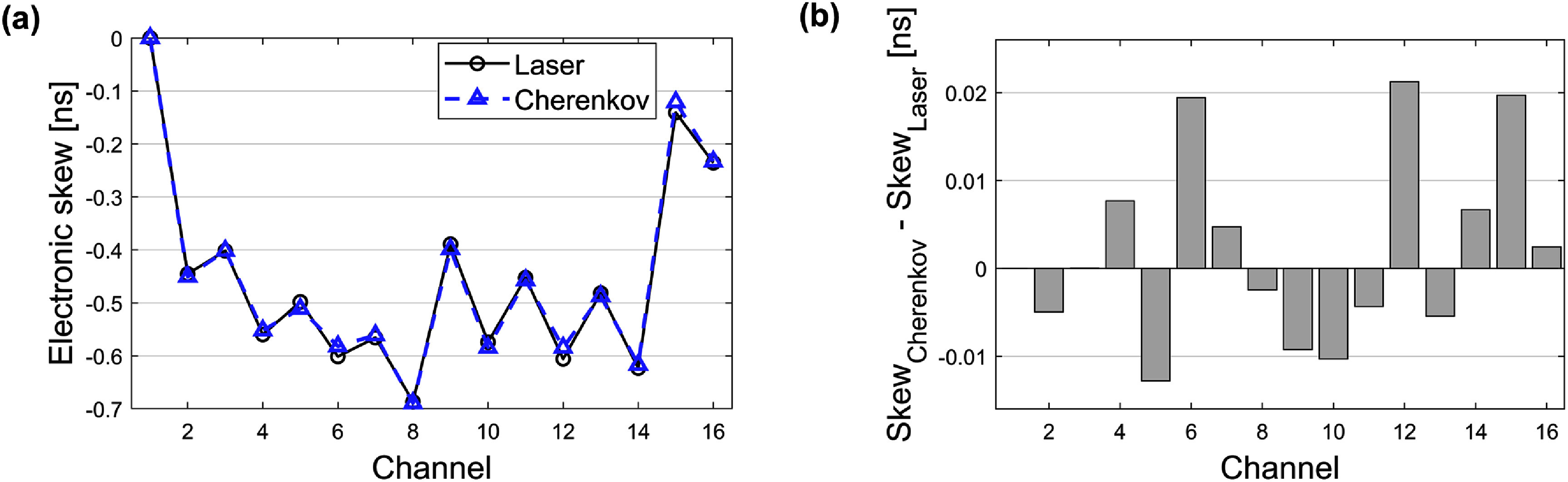
(a) Electronic skews relative to SiPM channel 1 measured from the laser setup and Cherenkov signature in FPDD and (b) differences between the two measurements.

Figure 7(a) shows the electronic time skews obtained from both the laser setup and the FPDD-derived method, normalized to 0 for channel 1. The two measurements showed close agreement across all channels, with a mean absolute error of 8 ps (figure 7(b)). This consistency validates the physical accuracy of the FPDDs, which leverage the capabilities for single Cherenkov photon detection and accurate event localization.

### 3.5. Time resolution

**Figure 8.**
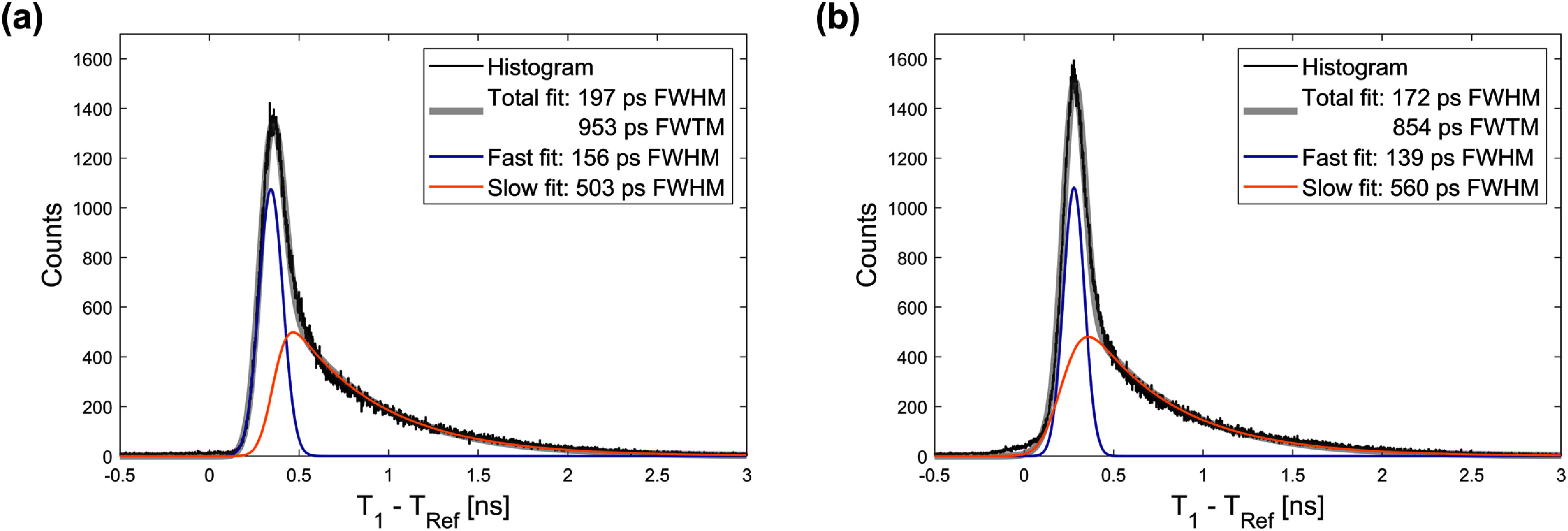
Time delay histograms (a) without and (b) with 3D position skew correction fitted with a sum of a single Gaussian (fast) and an exponentially modified Gaussian (slow).

Figure 8 shows delay histograms of the BGO detector timestamps relative to the reference detector, fitted with underlying Cherenkov plus early scintillation and slow scintillation components. Using the first photon timestamps, a distribution with 197 ps in FWHM and 953 ps in FWTM was observed, with 156 ps and 503 ps FWHM Gaussian and exponentially modified Gaussian components, respectively (figure 8(a)). Applying 3D skew corrections, derived from the prompt component of the FPDDs for each SiPM-voxel pair, improved the measured CTRs to 172 ps in FWHM and 854 ps in FWTM (figure 8(b)). The FWHM values for the Gaussian and exponentially modified Gaussian components comprising the fit to the skew corrected distribution were 139 ps and 560 ps, respectively. The estimated CTR between the fast distribution of two identical BGO slabs would be 186 ps FWHM. Events under the fast component, which accounted for 35% and 32% of total events without and with 3D skew correction, respectively, were more likely to be timestamped by Cherenkov photons and thus $\theta $ was primarily corrected for DOI effects. In comparison, events in the slower distribution contained a mixture of long transit Cherenkov photons and early arriving scintillation photons and were corrected with larger $\theta $ values to shift the timestamps toward the fast peak. As a result, the FWTM of the summed distribution was improved owing to the closer overlap between the two components. The ratios between FWTM and FWHM (FWTM/FWHM), 4.96 without correction and 5.12 with correction, were larger than those reported from studies on 3 × 3 × 20 mm^3^ BGO pixels (Lee *et al* 2025, Yi *et al* 2025). This is likely due to a combination of factors in our prototyping experimental setup, which we elaborate on in section 4.

### 3.6. Cherenkov probability-related parameters

**Figure 9.**
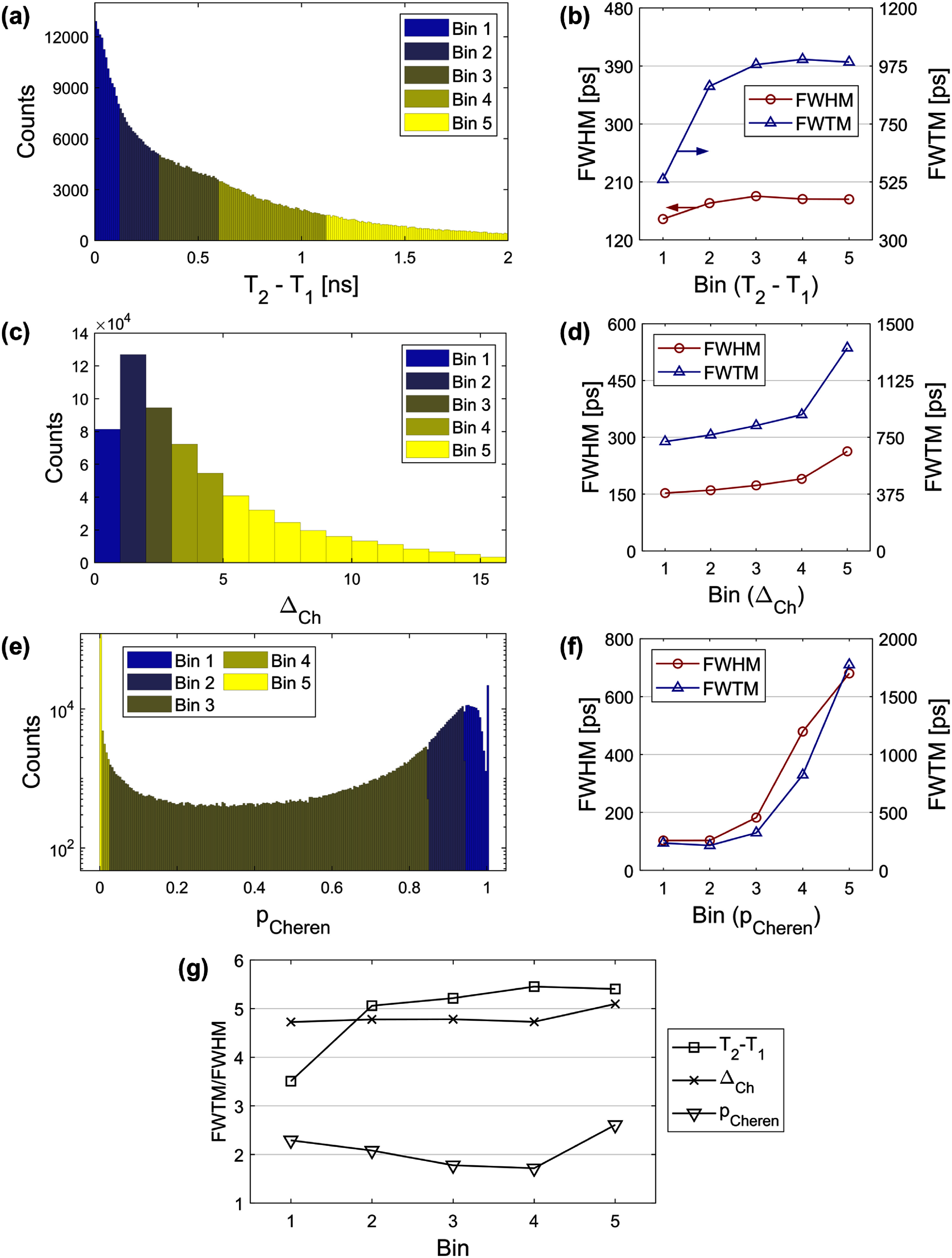
Analysis of candidate parameters that can correlate with Cherenkov detection probability. (a), (c), and (e) Distributions of T_2_–T_1_, Δ_Ch_, and p_Cheren_ with event binning, where lower bin numbers indicate a higher expectation of Cherenkov detection. (b), (d), and (f) Time resolutions in FWHM and FWTM for each bin. (g) FWTM-to-FWHM ratio of each bin classified by each parameter.

The distribution of *T*_2_–*T*_1_ is shown in figure 9(a) with event bins distinguished by color. Bin 1 contains 20% of events with the highest probability of involving Cherenkov detection, while Bin 5 contains the 20% with the lowest. The upper boundary of Bin 1 was 0.12 ns, and the total resolutions of 152 ps FWHM and 535 ps FWTM were achieved for events within this bin (figure 9(b)). Bins with larger *T*_2_–*T*_1_ showed not only degraded time resolution but also increased FWTM/FWHMs, indicating that the scintillation component dominated over the Cherenkov contribution (figure 9(g)).

Δ_Ch_, the measure of distance between the interaction position and the first photon arrival, also showed a correlation with the time resolution (figures 9(c) and (d)). The boundaries for Δ_Ch_ were defined to distribute the events across the bins as equally as possible. Both FWHM and FWTM improved for events with smaller Δ_Ch_, which resulted from the reduced likelihood of photons undergoing reflections and absorptions.

Figure 9(e) shows the distribution of *p*_Cheren_, the fraction of the Cherenkov component at the first-detected photon time point derived from the FPDDs. Events in Bin 5 are mostly characterized by *p*_Cheren_ = 0 due to the absence of a Cherenkov component peak at late timestamps, resulting in the poorest time resolution (figure 9(f)). In contrast, the time resolution improved with the higher *p*_Cheren_ events, achieving 103 ps FWHM and 237 ps FWTM with Bin 1.

FWTM/FWHM for each bin classified by three parameters are summarized in figure 9(g). This metric has been used to assess the dominance of the Cherenkov component in the time delay distributions of BGO detectors, with values approaching 1.8 when a single Gaussian fit well approximates the distribution due to suppression of the slow component (Kratochwil *et al* 2021a, Yi *et al* 2025). Among the parameters, *T*_2_–*T*_1_ yielded the most effective reduction in FWTM/FWHM, particularly for Bin 1, implying a stronger Cherenkov contribution in the corresponding events. In contrast, Δ_Ch_ exhibited minimal difference in FWTM/FWHM across the bins, whereas *p*_Cheren_ showed a distinct trend compared to the others.

## 4. Discussion

A prototype BGO semi-monolithic detector was characterized to investigate the spatiotemporal information available in a Cherenkov and scintillation photon counting detector and what potential advantages or opportunities such a capability might provide for BGO-based TOF-PET detectors. The custom 16-channel SiPM electronics featured a single photon pulse response of 1.8 ns on the fast outputs, enabling unique timestamping of individual optical photon arrivals. 69% of the 511 keV events in the BGO crystal were timestamped with the pure first photon signatures, i.e. TOT of the first digital pulse was one photon equivalent width (figure 3). The remaining ∼30% of events can be divided into two categories: (1) multiple Cherenkov/scintillation photon pileup and (2) single Cherenkov or scintillation accompanied by SiPM crosstalk (either direct and early external). Category 2) events still provide a unique, undisturbed timestamp of the primary photon. As the contribution of crosstalk was measured to be roughly 8% (section 3.2), we conclude that the first photon can be uniquely timestamped in 77% of all events. The remaining 23% corresponds to two or more optical photon pileups (category 1), which still physically includes the first photon, but the analog signal leading-edge slope becomes altered by subsequent photon arrivals in our detector configuration, which, in some cases, may lead to broadening the timestamp distribution used for time pickoff. The SPTR was measured to be 60 ps, confirming that the combined contributions of the intrinsic SPTR of the SiPM, the electronics, and the benchtop readout setup had only a marginal impact on overall timing performance. The detector was capable of event positioning with 3.17 mm resolution in the *X* direction using a CNN and 4-bin segmentation of DOI bins using the MM ratio metric. The transport dynamics of the first photon as a function of 3D interaction position were comprehensively characterized by parameterizing the Cherenkov and scintillation PDFs in the FPDDs. The usefulness of the parameter $\theta $ was demonstrated in both the accurate estimation of electronics skew (section 3.4.1) and 3D time skew for improving time resolution (section 3.5). In this study, we exploited a DOI-related metric, the MM ratio, to position events into DOI bins, which simplifies the calibration process. Further improvements in DOI estimation accuracy, as well as in the corresponding $\theta $ correction for timing, are expected with a full DOI calibration.

Exploiting the photon arrival time profiles and event position measured by this detector, we tested three different metrics, *T*_2_–*T*_1_, Δ_Ch_, and *p*_Cheren_, that are expected to encode the likelihood of an event to include Cherenkov signatures. All metrics showed reductions in both FWHM and FWTM for the bins with a higher expected Cherenkov contribution, with the strongest effect observed for *p*_Cheren_. Note that in this study, accurate estimation of *p*_Cheren_ was enabled by the use of a point source and a fast reference detector. The methodology for determining event-wise *p*_Cheren_ in arbitrary coincidence setups with extended sources is currently under development. Nonetheless, these preliminary results suggest that FPDDs can be used to quantify the probability of Cherenkov versus scintillation photon contributions. Bins 1 and 2, which together contained 40% of all events, achieved 100 ps FWHM. The FWTM-to-FWHM ratio, reflecting the mixture of fast and slow components in the time difference distribution between the detector and the reference, was used as a representative metric for Cherenkov detection probability, consistent with prior work (Kratochwil *et al* 2021a, Yi *et al* 2025). Among the tested metrics in this study, *T*_2_–*T*_1_ correlated with improved FWTM/FWHM ratio, while the behaviors of Δ_Ch_ and *p*_Cheren_ were not consistent with FWTM/FWHM reduction despite the improved FWHM and FWTM in Cherenkov-enriched bins. The unique FWHM and FWTM of each bin can be used to model adaptive TOF kernels to better address the probability of Cherenkov detection of each event, consequently enhancing the signal-to-noise ratio of the reconstructed images (Efthimiou *et al* 2021, Nuyts *et al* 2023). These classifiers should be further investigated to search for relevant parameters that better explain their physical correlation with Cherenkov detection probability, and by conducting simulations to quantify their Cherenkov and scintillation classification accuracy and to better understand the optical transport characteristics.

Compared to our previous study on a 16-channel monolithic BGO prototype detector, several advancements in both the readout electronics and overall detector performance have been achieved (Cates *et al* 2024). First, SiPMs with smaller active areas were used to leverage their superior intrinsic SPTR, owing to the smaller term2.6inal capacitance. A newer SiPM technology with reduced optical crosstalk also enabled applying higher SiPM bias voltages, thereby improving both PDE and SPTR (Merzi *et al* 2023, Lee *et al* 2025). Additionally, incorporating fast comparators in the timing chain allowed for increasing RF amplifier gain that was limited by the dynamic range of V1742 digitizers, and also mitigated deterioration in signal frequency response due to the limited bandwidth of V1742. Consequently, the semi-monolithic detector could fully exploit the excellent SPTR of the readout chain. Although a direct comparison is difficult by differences in crystal dimensions and SiPM arrangements, the 20 mm-thick semi-monolithic detector outperformed the 12 × 12 × 15 mm^3^ monolithic detector in CTR (186 ps versus 315 ps for the fast components of the identical detector pair) owing to the improved SPTR (60 ps versus 133 ps) and finer segmentation of interaction position (3.17 × 2 × 5 mm^3^ versus an estimated 4 × 4 × 7.5 mm^3^). We observed a lower fraction of events in the ‘fast’ Gaussian distribution in our prototyping experimental measurements conducted in this work (32% versus 51%). This is attributed to the following factors: (1) the reflective properties of the solid Teflon structure machined to house the BGO crystal (figure 2(a)) are not equivalent to optical grade Teflon tape, and its surface properties were not investigated; (2) the number of Cherenkov photon reflections is increased by high aspect ratio of the semi-monolith used in this work; (3) the linear array of 2 mm SiPMs assembled for this demonstration had relatively large, 0.68 mm gaps between their active regions, resulting in a lower fill factor (75% versus 81%). These factors limit the light collection efficiencies of both Cherenkov and scintillation, but are particularly critical for preserving low Cherenkov counts. Also, adding a 1 mm-thick optical bandpass filter absorbs an estimated 25% of Cherenkov light between 300 and 700 nm, which significantly reduces the Cherenkov yield. Ongoing work is evaluating the tradeoff between external crosstalk suppression and Cherenkov transmission with thinner glass filters.

**Figure 10.**
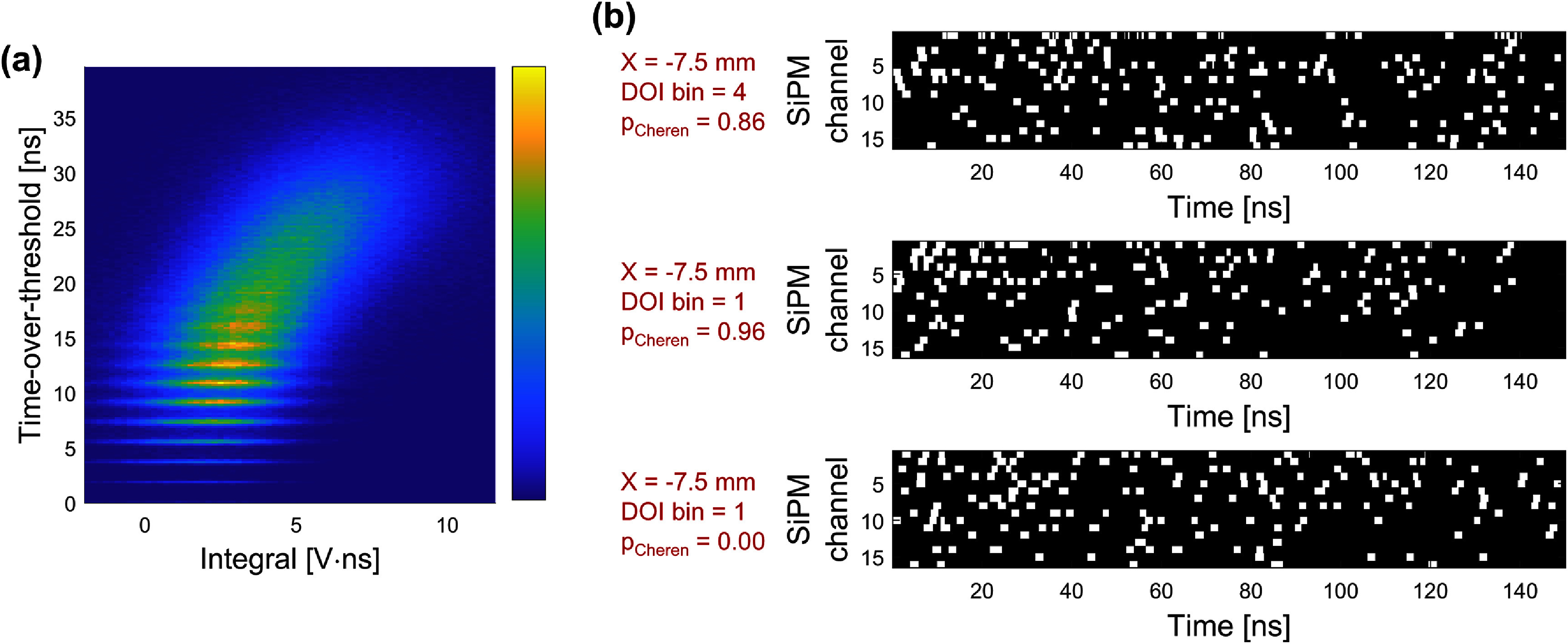
Illustrations of implementation possibilities of a fully time-based readout approach for photon counting detectors. (a) Correlation between the energy signal integral and summed TOT of the timing signal. (b) Example bitstreams generated from the sampled waveforms.

To pursue practical implementation of the photon counting technique in TOF-PET, a fully time-based readout scheme is in development. Conversion of the photon arrivals into the binary bitstream is conceptualized in figure 10(a), emulating the LVDS output waveform sampled at 5 GSa s^−1^ for 150 ns starting from the first timestamp (*T*_1_), in this work. This digitization scheme would enable access not only to the leading edges but also to later photon timestamps, which could be leveraged for further advancements in timing estimators. Energy estimation was performed using separate energy output channels, though this could be replaced by integrating the TOTs over time for each channel. Figure 10(a) shows the integral of energy signals versus the summed TOTs across all channels. Although the sampling window of the fast output waveform was limited to 200 ns in the current setup, which is insufficient to capture the full BGO emission profile, a positive correlation was observed with *R*^2^ = 0.6. Extending the sampling window and refining the event positioning algorithm are expected to further improve the position resolution while eliminating the need for additional analog-to-digital converters to process energy signals.

In this study, we focused on utilizing the earliest photon arrival timestamps on each channel to estimate the interaction time, primarily relying on *T*_1_. As illustrated in figure 10(b), however, a unique strength of this photon counting detector is its ability to extract rich spatiotemporal information from the individual photons. Each white speck represents a timestamp encoded by the rising edge and the corresponding photon count conveyed by the duration between the rising and falling edges, i.e. the TOT. Example events with an estimated X of −7.5 mm (perpendicular to SiPM channel 6) exhibited different patterns depending on DOI and *p*_Cheren_. For the event in DOI bin 4, photon arrivals were highly localized near the first detection channel within the first 5 ns. Among two events with the DOI bin of 1, the event with higher *p*_Cheren_ showed concentrated arrivals near the interaction, while the other detected the first photon apart from the interaction and showed relatively dispersed later photons over both time and the SiPMs.

Likewise, the rich information provided by the video-like frames of photon arrivals offers a wide range of applicable data-driven approaches to further improve the detector performance. For example, the *T*_2_–*T*_1_ metric used in this study is closely related to the metrics studied in previous works to categorize the events by the likelihood of being triggered by a Cherenkov photon, and thus can be used for time-walk correction (Kratochwil *et al* 2020, 2021a, Yi *et al* 2025). Interpreting the photon bitstream as a spatially extended waveform sampled across multiple channels also opens the possibility for a waveform-based approach to be implemented in this detector (Loignon-Houle *et al* 2025). In this study, FPDD provided a useful platform not only for linking the first photon arrival time to the spatial relationship between the interaction and detection but also for deriving separate PDFs for Cherenkov and scintillation detections, thereby allowing for the preliminary quantification of *p*_Cheren_ as a fraction of the Cherenkov PDF. The use of time delay distributions can be extended to the later photons to more accurately integrate the photon transport dynamics into the estimation of interaction time and Cherenkov detection probability, particularly for recovering photon arrivals affected by complex travel paths within a large crystal volume.

Building on the foundations with the 1D detector investigated in this study, the next step is to scale up to a full detector module to increase detection coverage. The module will comprise stacked BGO slabs and a larger SiPM array, requiring the development of signal multiplexing techniques that reduce the number of SiPM readout channels while preserving the information extracted in these studies. A precise, scalable data acquisition setup will be constructed and examined to reproduce the performance achieved from the waveform-sampling benchtop setup used in this study. A similar position and time calibration framework can be employed, though automated procedures are required to ensure scalability and uniformity at the scanner level. An ultimate goal would be to employ techniques similar to those that translate the calibrations from one detector to an entire system by training a neural network to learn the association of the detector calibrations with a particular attribute, which can be gathered from a simple flood irradiation (Freire *et al* 2021). These methods have made semi-monolithic crystal calibrations tractable for an entire total-body TOF-PET system. Integrated into a full system, accurate estimation of Cherenkov detection probability could enable multi-TOF-kernel image reconstruction, improving image performance with the same scan time. In the long term, the rich information extracted from the proposed photon counting approach, in combination with detector designs or technologies that enhance the collection of Cherenkov light, has the potential to improve TOF precision and sensitivity in BGO-based PET scanners. The present work suggests the potential to approach 100 ps FWHM CTR with thick BGO slabs with adequate Cherenkov detection efficiency, as shown in figure 9(f), with coincidence distributions that do not exhibit long tails from timing estimators derived from delayed scintillation light (figure 9(g)). Since SPTR plays a strong role in this detector configuration (Gundacker *et al* 2023), the achievable CTR will also continue to improve as photosensor technologies improve, as our approach also allows for the correction of scintillation photon transit time delay. In addition, there are advanced timing estimators to explore that combine the coupled 3D spatial and timing information from all available photon statistics to simultaneously estimate time and position of interaction. Altogether, the work presented here shows the potential for BGO to advance TOF-PET with photon counting detectors and signal processing methods that label Cherenkov and scintillation light.

## 5. Conclusion

This study demonstrated the Cherenkov and scintillation photon counting capabilities of a prototype semi-monolithic BGO detector with 16-channel SiPM readout electronics. By leveraging the fast timing response of the SiPMs and LNHF electronics and 3D event positioning information, we achieved a high probability of the first photon to be uniquely timestamped and performed transport time corrections for Cherenkov and scintillation photons via FPDDs. This 20 mm thick slab of BGO achieved 172 ps FWHM in coincidence with a fast reference detector and 186 ps FWHM for the fast component of the BGO pair by correcting the first photon detection time for its dependency on interaction position. We also performed an exploratory analysis of parameters correlated with Cherenkov detection probability and time resolution, which may enable event labeling and correction. The promising, data-rich characteristics of the detector shown in this study lay the foundation for a high-sensitivity and performance TOF-PET detector by integrating photon counting capabilities with data-driven Cherenkov identification techniques.

## Data Availability

All data that support the findings of this study are included within the article (and any supplementary information files).
